# Carotid Intima-Media Thickness in Asymptomatic Subjects With Low Lipoprotein(a) Levels

**DOI:** 10.4021/jocmr849w

**Published:** 2012-03-23

**Authors:** Kazuhiko Kotani, Naoki Sakane

**Affiliations:** a Division of Preventive Medicine, Clinical Research Institute, National Hospital Organization Kyoto Medical Center, Kyoto, Japan; b Department of Clinical Laboratory Medicine, Jichi Medical University, Tochigi, Japan

## Abstract

**Background:**

Elevated and extreme circulating levels of lipoprotein(a) (Lp(a)) are considered to be an atherosclerotic risk factor, although additional studies on the low levels of Lp(a) are necessary to provide confirmation. The carotid intima-media thickness (CIMT) is known as a surrogate index of atherosclerosis. The aim of the present study was to investigate the correlation between the serum Lp(a) and CIMT in asymptomatic subjects with a relatively low Lp(a) level.

**Methods:**

The study included 65 asymptomatic female Japanese subjects (mean age: 60 years) with a serum Lp(a) level < 30 mg/dL. Clinical data including the serum Lp(a) and CIMT were measured, and their correlations were examined.

**Results:**

The median Lp(a) level was 18.6 mg/dL and the mean CIMT level was 0.8 mm. There was a significant and inverse correlation between the CIMT and Lp(a) (r = - 0.24, P ≤ 0.05), in addition to a significant and positive correlation between the CIMT and subject age and systolic blood pressure. A stepwise multiple linear regression analysis identified the Lp(a) to be correlated independently, significantly and inversely with the CIMT.

**Conclusions:**

The Lp(a) levels were inversely correlated with the CIMT in this population, suggesting that subjects with a low Lp(a) level may have a predisposition to carotid atherosclerosis. This finding was preliminary and should be investigated further in larger studies and in additional settings.

**Keywords:**

Lp(a); Carotid artery; IMT; Atherosclerosis

## Introduction

Elevated, and particularly extreme, circulating levels of Lp(a) are considered to be a risk factor for cardiovascular disease (CVD) [1-3]. The dual structure of Lp(a), consisting of a cholesterol-laden low-density lipoprotein (LDL) particle and a plasminogen-like glycoprotein apolipoprotein(a), implies that Lp(a) can contribute to both atherosclerosis and thrombosis [2, 3]. In fact, Lp(a) has been observed to accumulate in arterial lesions [4]. However, at present, the role of Lp(a) in the pathogenesis of atherosclerosis has not been completely elucidated, so the identification of various factors that affect the association of Lp(a) with atherosclerotic manifestations is necessary.

The sonographic measurement of the thickness of the carotid artery, the carotid intima-media thickness (CIMT), is one of the best accepted surrogate indices reflecting not only local, but also generalized atherosclerosis, associated with cardio- and cerebro-vascular morbidity and mortality, even in asymptomatic individuals [5]. However, the associations observed between the Lp(a) and CIMT have been inconsistent, with several studies having reported positive associations [6-15], while other reported no significant associations [16-24]. There was also a report showing that there was an association between the Lp(a) and CIMT only in the severe state of the disease [25].

The circulating Lp(a) concentrations vary very widely among the populations, and there is generally a skewed distribution, with a high prevalence of the low concentrations [26-28]. In the Japanese population, the distribution of Lp(a) shows skewing toward the lower concentrations, with most of the population having concentrations < 30 mg/dL, which is considered to be a presumed normal high level of Lp(a) [1]. However, no clinical studies have specifically examined subjects with a low Lp(a) level. We have noted the implications of a low Lp(a) level in two studies [16, 29]. In those studies, subjects with high CIMT levels were not rarely observed among those with low Lp(a) levels in one study [16] and the J-curve phenomenon for the incidence of CVD (an increase of the incidence in subjects with the lowest Lp(a) levels) was observed [29]. These findings may be partly related to inconsistent results found for the association between the Lp(a) and CIMT [15-25]. Therefore, the present study was aimed to investigate the correlation between the serum Lp(a) and CIMT in asymptomatic subjects, restricted to those with a low Lp(a) level.

## Methods

A total of 65 female Japanese subjects were consecutively recruited from our outpatient clinics. Eligible subjects were asymptomatic, non-pregnant and not taking any medications. Only subjects with a serum Lp(a) level < 30 mg/dL were included. Subjects who were regular drinkers, and those with a history of CVD, malignancy, endocrine disorders, or severe kidney and liver diseases were excluded. The institutional ethics committee approved the study, and each subject gave informed consent.

Current smoking habits were self-reported. The body mass index (BMI), seated systolic blood pressure (SBP)/diastolic blood pressure (DBP) in the upper arm, and the plasma glucose, serum lipids and Lp(a) levels were measured after an overnight fast. The glucose and lipids (TC: total cholesterol, HDL-C: high-density lipoprotein cholesterol and TG: triglycerides) were measured enzymatically, and the serum Lp(a) was measured by an ELISA system (Sekisui Medical Co. Ltd., Tokyo, Japan). The CIMT of the common carotid arteries was measured ultrasonographically by a 10 MHz linear type B-mode probe (Xario system: Toshiba Co. Ltd., Tokyo, Japan). The CIMT, bilaterally measured in segments free of plaque (one at the thickest site and another at two other points (1 cm upstream and 1 cm downstream from the thickest site)), was averaged for 3 measurements.

The data are expressed as the means ± standard deviation or medians plus interquartile range. The correlations between the CIMT and the other variables, including the Lp(a), were examined by Pearson's correlation test, and subsequently, a stepwise multiple linear regression analysis (with F for the entry set to 2) adjusted for the measured variables (age, smoking, BMI, SBP, DBP, TC, HDL-C, TG, glucose). In these analyses, the TG and Lp(a) values were calculated after a log-transformation because of their skewed distributions. A P-value ≤ 0.05 was considered to be significant.

## Results

The clinical characteristics of the study subjects are listed in Table 1. The simple correlations between the CIMT and other variables, including the Lp(a), are listed in Table 2. There was a significant and positive correlation between the CIMT and subject age or SBP, while a significant and inverse correlation between the CIMT and Lp(a) was observed.

**Table 1:** Clinical Characteristics of Study Subjects

Variables	Levels
Age, years	60 ± 10
Current smoking, n (%)	9 (14%)
Body mass index, kg/m^2^	23.6 ± 3.6
Systolic blood pressure, mmHg	135 ± 19
Diastolic blood pressure, mmHg	78 ± 9
Total cholesterol, mg/dL	261 ± 38
HDL-cholesterol, mg/dL	68 ± 14
Triglycerides, mg/dL	125 (92 - 181)
Plasma glucose, mg/dL	107 ± 22
Lp(a), mg/dL	18.6 (8.9 - 24.7)
CIMT, mm	0.8 ± 0.2

HDL: high-density lipoprotein, Lp(a): lipoprotein(a). The values are expressed as the means ± standard deviation, medians (interquartile range) or numbers.

**Table 2:** Correlations Between the CIMT and Other Variables, Including the Lp(a), in This Population

Variables	r	P-value	β	P-value
Age, years	0.33	< 0.01*	0.32	< 0.01*
Current smoking, n (%)	0.06	0.66	Not extracted	-
Body mass index, kg/m^2^	0.06	0.65	Not extracted	-
Systolic blood pressure, mmHg	0.30	0.02*	Not extracted	-
Diastolic blood pressure, mmHg	0.17	0.17	Not extracted	-
Total cholesterol, mg/dL	0.05	0.67	Not extracted	-
HDL-cholesterol, mg/dL	- 0.12	0.33	Not extracted	-
Triglycerides, mg/dL	0.02	0.88	Not extracted	-
Plasma glucose, mg/dL	0.21	0.09	Not extracted	-
Lp(a), mg/dL	- 0.24	0.05*	- 0.23	0.05*

HDL: high-density lipoprotein, Lp(a): lipoprotein(a). r: simple correlation test (Pearson’s test), β: stepwise multiple linear regression analysis adjusted for all the listed variables. Significance level: * P ≤ 0.05.

A stepwise multiple linear regression analysis identified subject age to be correlated independently, significantly and positively with the CIMT, and the Lp(a) to be correlated independently, significantly and inversely with the CIMT in this population.

## Discussion

The present study showed that the Lp(a) levels were inversely correlated with the CIMT in asymptomatic female subjects within a Lp(a) < 30 mg/dL. This suggests that subjects with a low Lp(a) level may have a predisposition to carotid atherosclerosis. This study was conducted in a relatively small population, and the correlation was not very strong. However, if the results are confirmed in large studies, the present findings may provide new insights into the physiological roles of Lp(a) in atherosclerosis.

Of importance, a recent investigation has reported that when Lp(a) is present at not so high levels, it can function as a scavenger that absorbs oxidized lipids in the circulation and vessel walls [30]. Accordingly, some minimal level of Lp(a) may be necessary to protect against atherosclerotic progression, although this concept will need to be verified in additional clinical studies.The circulating Lp(a) levels are thought to be largely under genetic control (i.e., at the concentration of biosynthesis of the apolipoprotein(a), which is encoded by the *LPA* locus) [31]. A few studies have been conducted on the significant association among Lp(a) levels, Lp(a)-related gene polymorphisms and atherosclerosis [32, 33]. Although the evidence of the association of the gene polymorphisms relating to low Lp(a) levels in subjects with atherosclerosis remains insufficient, Lp(a) gene polymorphisms and/or its linkage equilibrium may be associated with carotid atherosclerosis. In addition, there was a report that showed a significant interaction of lifestyle factors (i.e., daily fish intake) and Lp(a)-related gene polymorphisms in decreasing the Lp(a) levels, although one would generally not consider lifestyle factors to have a major influence on the Lp(a) levels [34]. We did not obtain information about the full lifestyle-related factors of the present study subjects, but specific lifestyle-related factors may, at least in part, be associated with the results of the present study, from the previous study findings [34]. Future investigations, taking into consideration genetic and lifestyle factors, may be needed to clarify the biological importance of the results.

There are additional limitations to our study. The present study was relatively small and cross-sectional. Therefore, we could not conclusively determine the presence of any cause-and-effect relationship based on the results. Furthermore, our study population was asymptomatic and restricted to females and Japanese subjects. Future studies on symptomatic diseased patients and males or other ethnic groups are required.

In summary, this study found that the Lp(a) levels were inversely correlated with the CIMT in asymptomatic female subjects, with a serum Lp(a) level < 30 mg/dL. This suggests that subjects with a low Lp(a) level may have a predisposition to carotid atherosclerosis. This clinical finding should be further investigated in larger studies and additional settings.
